# Bolaamphiphiles Derived from Alkenyl L-Rhamnosides and Alkenyl D-Xylosides: Importance of the Hydrophilic Head

**DOI:** 10.3390/molecules18056101

**Published:** 2013-05-22

**Authors:** Sylvain Gatard, Mehmet Nail Nasir, Magali Deleu, Nadia Klai, Vincent Legrand, Sandrine Bouquillon

**Affiliations:** 1 Unité Mixte de Recherche “Réactions Sélectives et Applications”, CNRS—Université de Reims Champagne-Ardenne, Boîte n 44, B.P. 1039, F-51687 Reims, France; 2 Unité de Chimie Biologique Industrielle, Gembloux Agro-Bio Tech, Université de Liège, 2, Passage des Déportés, B-5030 Gembloux, Belgium; 3 Centre de Biophysique Moleculaire Numerique, Gembloux Agro-Bio Tech, Université de Liège, 2, Passage des Déportés, B-5030 Gembloux, Belgium

**Keywords:** rhamnose, glycosylation, metathesis, surfactant, bolaform, interfacial properties

## Abstract

The two step synthesis of a new bolaamphiphile derived from alkenyl L-rhamnosides was described. The general synthetic strategy of bolaamphiphiles derived from L-rhamnose was based on a previous work describing the synthesis of bolaamphiphiles derived from D-xylose. The conformational properties of this new compound were investigated by FTIR spectroscopy in an aqueous film in order to obtain a reference for further studies about the membrane-interacting properties. Moreover, the surface activity of this new bolaamphiphile was analyzed by Langmuir balance technology and was compared with that of the analogous bolaamphiphile derived from alkenyl D-xylosides. The findings indicate that the rhamnoside-based bolaform has an increased surface activity and a better ability to form aggregates than xyloside-based one.

## 1. Introduction

Sugar-based biosurfactants are considering as environmental-friendly molecules due to their biocompatibility and biodegradability. Their use has thus become more widespread in recent years because of environmental and toxicity issues [1]. In this context, our work was focused on a special class of sugar-based surfactant derived from L-rhamnosides which could be considered as rhamnolipids. Rhamnolipids constitute a class of glycolipid known as the best characterized of the bacterial surfactants [2,3,4,5]. In particular, rhamnolipids have been broadly used in the cosmetic industry for products such as moisturizers, toothpaste, condom lubricant and shampoo [2]. Rhamnolipids are efficient in bioremediation of organic and heavy metal polluted sites [6]; they have also long been reported to have antimicrobial properties [7] and to have plant defence elicitor activities [8,9]

**Figure 1:**
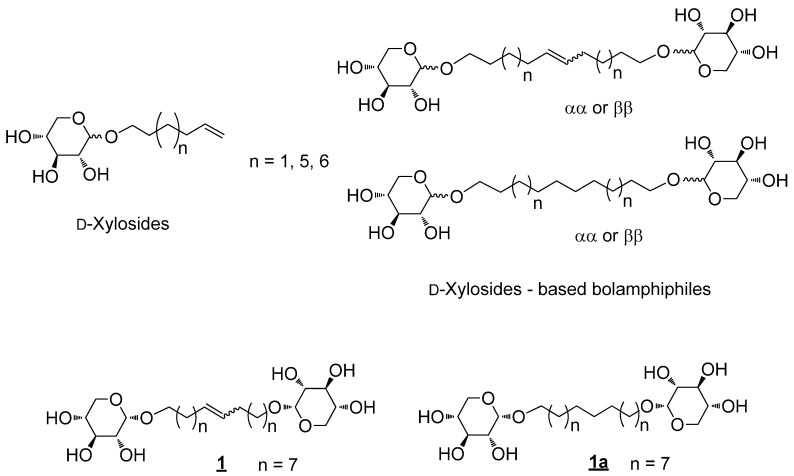
D-xylosides and D-xylosides-based bolaamphiphiles.

We previously described the synthesis and the interfacial and membrane properties of D-xylosidesand D-xylosides-based bolaamphiphiles (Figure 1) [10,11,12].

In these last studies, the monolayer properties, the adsorption behavior and membrane destabilization properties of two D-xylosides-based bolaamphiphiles differing by their spacers (presence or absence of one double bond on a C18 chain, Figure 1) were investigated. The presence of one unsaturation has no influence on the interfacial organization at low compression but impairs the stability of the monolayer at high compression [10,11,12].

In the present study, we focused on a rhamnoside-based bolaamphiphile differing from xylose-based ones only by containing L-rhamnoside instead of D-xyoside at the level of the hydrophilic heads. L-rhamnose was used as starting sugar to synthetize by metathesis L-rhamnoside-based bolaamphiphile with a C_18_ unsaturated chain. Its conformational and surface-active properties were analysed. These properties, susceptible to play a crucial role on the interactions with biological membranes, were compared with those of the analogous D-xylosides-based bolaamphiphile in order to evaluate the role of the hydrophilic head. 

## 2. Results and Discussion 

### 2.1. Synthesis of the 1', 18'-bis-octadec-9'-enyl-α-L-rhamnopyranoside (**3**)

**Scheme 1:**
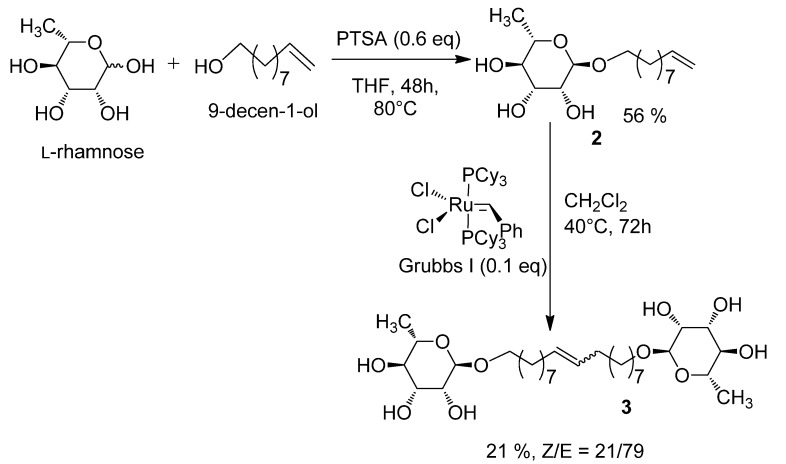
Synthesis of bolaamphiphile **3**.

The general synthesis strategy was based on previously described work concerning bolaamphiphiles derived from D-xylose [10]. In a first step, a glycosylation of the L-rhamnose with 9-decen-1-ol following Fischer’s method [12] led to a rhamnoside with very good stereoselectivity in favour of the monocatenar α (ratio α/β: 95/5) as typically described for other glycosylation of L-rhamnose related in the literature [13,14,15]. In a second step, classical conditions of metathesis reaction [10] in the presence of a catalytic quantity of Grubbs I catalyst gave the bolaamphiphile **3** with a yield of 21% and a *Z/E* ratio around 20/80 (Scheme 1).

Unlike the synthesis from D-xylose [10] the glycosylation of L-rhamnose with 9-decenol is stereospecific because only the α anomer is obtained, as confirmed by a single signal at 101.6 ppm in the ^13^C-NMR spectrum. This reactivity was already described by Jung [16]. This makes easier the preparation of the corresponding stereospecific bolaamphiphile αα by suppressing the protection/deprotection steps generally required for the separation of different anomers. The direct metathesis of the L-rhamnoside **2** led to a 21% yield of the bolaamphiphile **3** as a classical 20/80 mixture of *Z/E* isomers. This low yield could be explained because of the high viscosity of the compound **3**, obtained as a relatively difficult to purify wax. Further improvement of the purification steps, especially the one concerning the elimination of the metallic residue is required to increase the yield. 

### 2.2. FTIR Spectroscopy 

FTIR spectroscopy is a very sensitive technique to the presence of chemical groups and the hydrogen bonds. After the synthesis, we performed the FTIR measurement of the bolaamphiphile **3** in solid state in order to examine if the expected chemical groups are present within the molecule. In a second experiment, before starting the physicochemical characterization, we performed the FTIR measurement of the D_2_O-hydrated film of the bolaamphiphile **3**. This spectrum of the D_2_O-hydrated film was compared to those obtained in solid state for detecting chemical groups which can be involved in hydrogen bonding and will serve as reference for further studies of interaction of this molecule with biological membrane lipids. 

**Figure 2:**
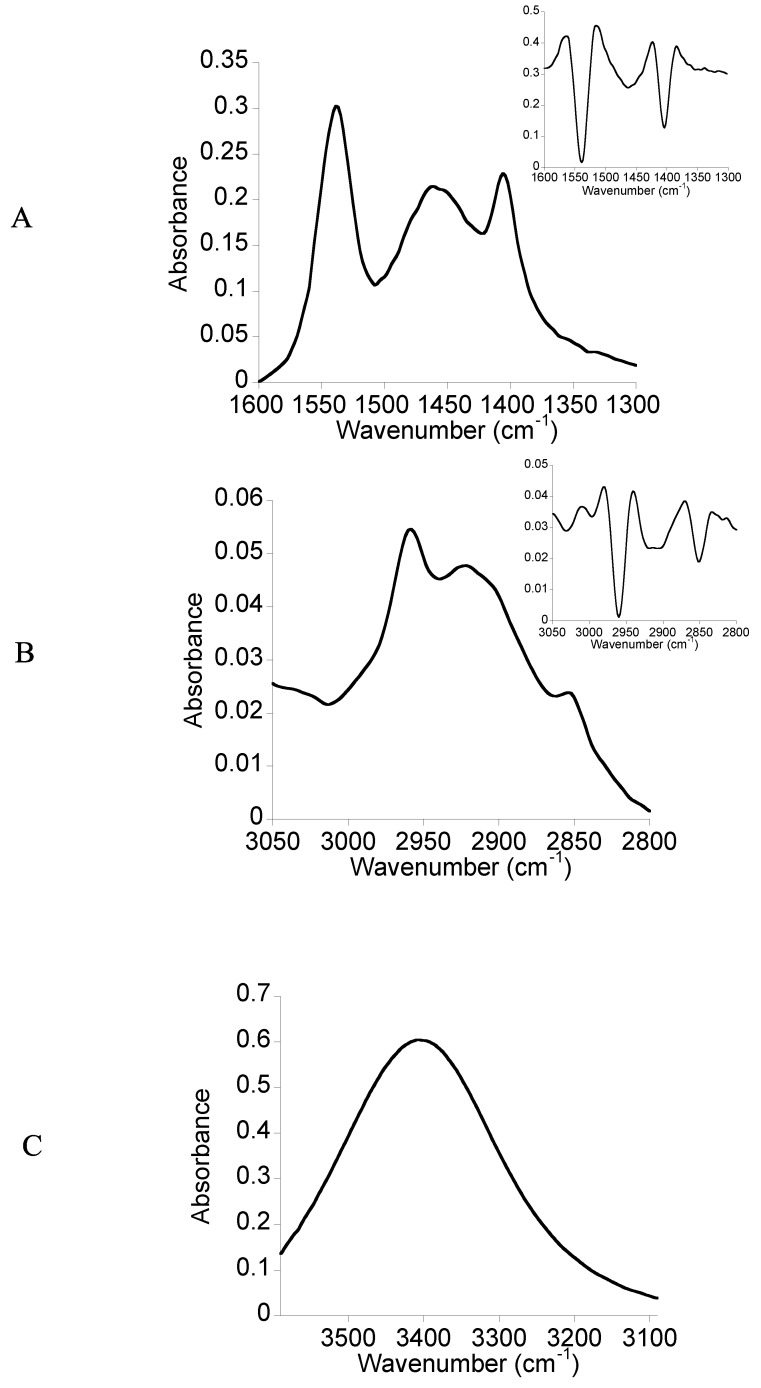
FTIR spectra of the D_2_O-hydrated film of the bolaamphiphile **3**. (**A**) The 1,600–1,300 cm^−1^ region; (**B**) the 3,050–2,800 cm^−1^ region; (**C**) the 3,600-3,090 cm^−1^ region.

The Figure 2 gives the three regions of FTIR spectrum of the D_2_O-hydrated film of the bolaamphiphile **3**. In the 1,600–1,300 cm^−1^ region of the FTIR spectrum (Figure 2-A), three bands are observed. The second derivative spectrum (insert Figure 2-A), processed to better distinguish the wavenumber of the bands gives three peaks at 1,537, 1,458 and 1,403 cm^−1^. The peak at 1,537 cm^−1^ could be attributed to the residual presence of CH_2_Cl_2_ which became more significant in the case of hydrated films. The peak at 1,458 cm^−1^ could be attributed to the bending vibrations of CH_2_ groups of the hydrocarbon chain and the peak at 1,403 cm^−1^ to the OH bending vibrations in the plane of the alcohol groups of two L-rhamnosides. 

Figure 2-B shows the 3,050–2,800 cm^−1^ region. The second derivative of the spectrum displayed three peaks located at 2,958, 2,913 and 2,851 cm^−1^. The absorbance in this region corresponds to the stretching vibrations of the CH_2_ and CH_3_ groups of the hydrocarbon chain. 

Figure 2-C corresponds to the 3,590–3,090 cm^−1^ region of the spectrum. A broad band located at 3,396 cm^−1^ is observed. It corresponds to the alcohol groups of two L-rhamnosides. Compared to the spectrum of the solid state the shift to lower wavenumber and the broad shape could be attributed to the presence of alcohol groups involved in hydrogen bonds.

### 2.3. Surface Activity of the Rhamnoside-Based Bolaform (Bolaamphiphile **3**)

**Figure 3:**
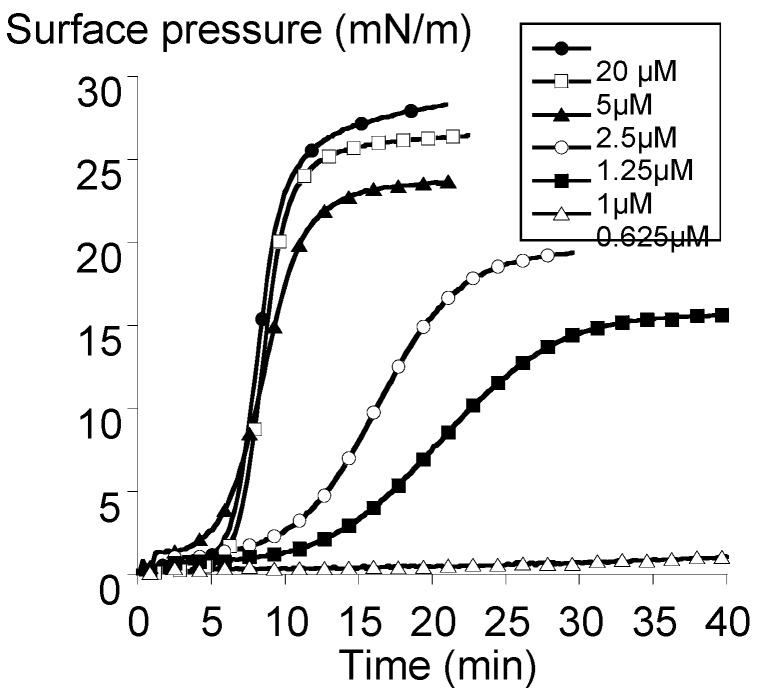
Surface activity of the bolaamphiphile **3**. Kinetics of bolaform adsorption at the air-water interface. Time zero corresponds to the injection of the bolaform into the subphase.

The surface pressure is the difference between the tension of the neat air-water interface and the tension of the air-water interface in the presence of a molecular film. The kinetics profiles of the surface pressure at various concentrations of the bolaamphiphile **3** were examined in order to analyze its abilities to form an interfacial film (Figure 3). 

For concentrations less than 0.5 µM, no bolaform-induced increase of the surface pressure is observed (data not shown). This could indicate that bolaamphiphile **3** cannot adsorb to the air-water interface or even though there was an adsorption, the coverage of the surface was not sufficient for detecting an increase of the surface pressure [17]. For concentrations equal or greater than 0.625 µM, whatever the concentration of bolaamphiphile **3** injected into the subphase, we observed a sigmoïdal increase of the surface pressure indicating that the adsorption of the bolaamphiphile over time was gradual and reached a steady-state more or less rapidly depending on the concentration. Moreover, there was a lag period prior to the detection of the increase of the surface pressure depending on the concentration of the bolaform into the subphase. The difference between lag periods was more significant for concentrations between 0.625–2.5 µM and became less important for 2.5–20 µM. This shows that the more bolaform concentration into the subphase is, the faster the adsorption of the bolaform to the air–water interface is. 

Suggesting that increasing the amount of bolaform molecules into the subphase increased the probability of the interactions between them, it could be proposed that these interactions played an important role on the adsorption of bolaform molecules at the air-water interface as in the case of other surface-active molecules [17]. This interpretation seems to be also consistent with the absence of the increase of the surface pressure when the concentration of the bolaform in the subphase did not reached the threshold concentration for having enough interactions between them as previously proposed by Volinsky *et al.* or Sun *et al.*[18,19].

**Figure 4:**
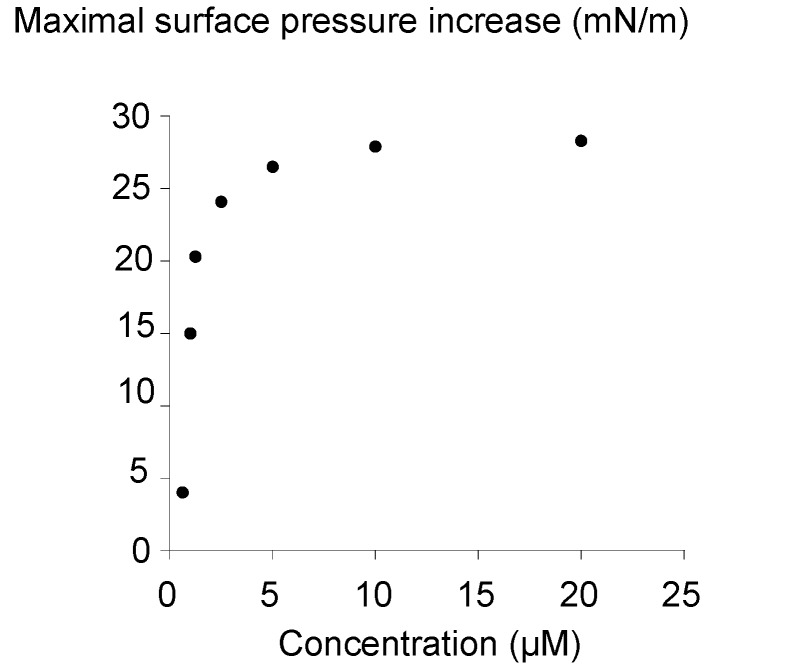
Influence of the bolaamphiphile **3** concentration on the reached maximal surface pressure.

Figure 4 shows the maximal surface pressure induced by the bolaform adsorption as a function of the bolaform concentration in the subphase. The relationship between the amount of the bolaform molecules adsorbed at the air-water interface and the concentration of the bolaform molecules in the subphase is not linear. 

After reaching a threshold concentration, there was no increase of the surface pressure even though the concentration into the subphase increased. This break could indicate a saturation of the interface. This threshold concentration could correspond to a state where the interactions between bolaform molecules were strong enough to cover the entire available surface. These interactions could occur in the direction of the surface between molecules and conceptualized as “work of adhesion” [20]. The interactions in the direction of the surface at this threshold concentration would be a key factor for the surface coverage. Above this concentration, aggregates of bolaform in the bulk phase should be formed. From this curve, the critical aggregation concentration (CAC) of the bolaamphiphile **3** could be determined at the intersection of two linear fittings of the curve. The CAC of bolaamphiphile **3** is 2.3 ± 1.4 µM and the corresponding γ_CAC_ is 49 ± 1.9 mN/m.

**Figure 5:**
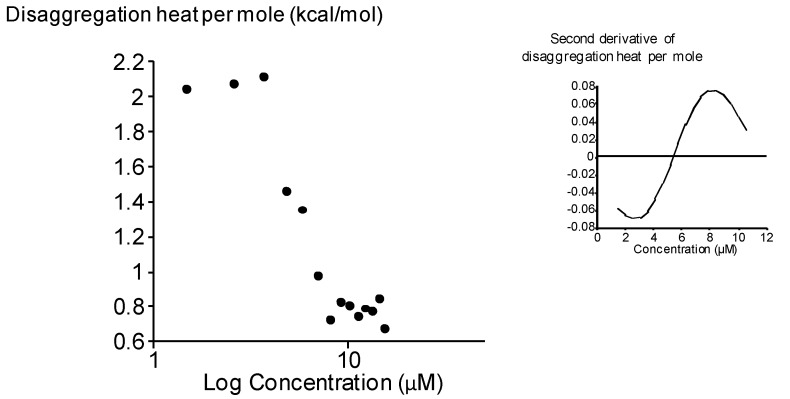
Critical aggregation concentration (CAC) of bolaamphiphile **1** determined by ITC measurements. The graph show the heat of disaggregation per mole of **1** plotted versus the concentration logarithm of the bolaform in the ITC cell. Inset: second derivative of the disaggregation heat as a function of concentration.

In a previous study, the CAC of the analogue bolaamphiphile **1 **derived from D-xylose differing in the hydrophilic heads (xylose instead of rhamnose) was determined by ITC measurements as 50 µM [10]. This value is not in the same range as that obtained in the present study. New ITC measurements were then performed in a range of lower concentrations. The results revealed the existence of another aggregation state. The Figure 5 gives the heat of disaggregation per mole of bolaamphiphile **1** (δhi/δn**1**) as a function of its concentration in the measurement cell. This sigmoidal curve shows that the (δhi/δn**1**) decreased when the concentration of bolaamphiphile** 1** increased. The principle of the determination of the CAC by ITC is based on the relationship between the number of molecules in the solution and the energy released upon break-up of their aggregates (δhi). Every injection into the sample cell increases the number of compound **1** in the solution and decreases the δhi until a threshold value approaching zero. The determination of the inflection point of this sigmoidal curve (by the second derivative, see inset) gave the CAC value which is 5.6 ± 0.4 µM for bolaamphiphile **1**. 

This value was at the same range than the one obtained for bolaamphiphile **3** in the present study. These findings could be interpreted as the existence of two states of aggregation for bolaamphiphile **1**. The first state at 5.5µM could correspond to a state for which a molecular association between xyloside-based bolaforms to form a micelle-like structure while the second state at 50 µM could agree with a supramolecular association of the aggregated structures. This second step was not observed for bolaamphiphile **3**.

If we considered the first step of aggregation, the CAC of the bolaamphiphile **3** was slightly less than those obtained for bolaamphiphile **1** showing that the bolaamphiphile **3** is more able to form aggregates than bolaamphiphile **1**. Then, we can conclude that the hydrophilic heads of the bolaform play an important role on their capacity of aggregation. The importance of the polar head on the aggregation behavior of the surfactants has been previously shown in the case of N-methyl, N-polyethyleneglycol dodecylamide type surfactants. These authors have reported that the increase of the head group polarity results into an increase of the CAC value [21]. Our results are in accordance with this study. Indeed, the presence of a supplementary methyl group on the hydrophilic head of the bolaamphiphile **3** compared to the bolaamphiphile **1** increases the global hydrophobicity of the molecule which seems in favour of an aggregation. 

## 3. Experimental

### 3.1. General

All reagents were commercially available and used as received. Solvents were dried and distilled under argon before use (CH_2_Cl_2_ over CaCl_2_ and diethyl ether, THF over sodium/benzophenone) and stored over molecular sieves. ^1^H- (250.1 MHz) and ^13^C-NMR (62.9 MHz) spectra were recorded on an AC 250 Bruker instrument in CDCl_3_, MeOD or acetone-*d*_6_ with TMS as reference for ^1^H spectra and CDCl_3_ (δ 77.0), MeOD (δ 49.9) or acetone-*d*_6_ (δ 30.6) for ^13^C spectra. The infrared spectra were recorded with Spectrafile IR^TM^ Plus MIDAC. C, H and N analyses were performed on a Perkin Elmer 2400 CHN equipment. GC was recorded on a Hewlett-Packard HP-6890 gas chromatograph, fitted with DB-1 capillary column (25 m, 0.32 mm), a flame ionisation detector and HP-3395 integrator; chromatography was carried out on SDS Silica 60 (40–63 μm), Art 2050044 (flash-chromatography) or Silica 60 F_254_ (TLC plates). All experiments (MS and HRMS) were obtained on a hybrid tandem quadrupole/time-of-flight (Q-TOF) instrument, equipped with a pneumatically assisted electrospray (Z-spray) ion source (Micromass, Manchester, UK) operated in positive mode. The electrospray potential was set to 3 kV in positive ion mode (flow of injection 5 μL/min.) and the extraction cone voltage was usually varied between 30 and 90 V.

*Dec-9-enyl-α-rhamnopyranoside* (**2**)


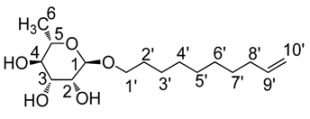


To a solution of L-rhamnose (4 g; 24 mmol; 1 eq) and 9-decen-1-ol (8.6 mL; 48 mmol; 2 eq) in THF (20 mL) are added at 80 °C, 2.7 g of PTSA (14 mmol; 0.6 eq) in three portions (900 mg each h). After 48 h of reaction, the mixture is neutralized with addition of a 0.5 M MeONa solution (≈20 mL) and the purification of the major α anomer (ratio α/β: 95/5) is realized through flash chromatography (eluting mixture: CH_2_Cl_2_/MeOH 9:1). Compound **2** is obtained as a brown paste with a yield of 56%. IR (KBr) ν cm^−1^: 3407 (F), 2928 (F), 2856 (m), 1640 (f), 1456 (f), 1132 (m), 1056 (F). ^1^H-NMR (CD_3_OD): δ (ppm) 1.23 (d, 3H, *J* = 7.5 Hz, H6), 1.32 (br, 10H, H3', H4', H5', H6', H7'), 1.56 (br m, 2H, H2'), 2.03 (br m, 2H, H8'), 3.35 (overlap, 2H, H1'a, H4), 3.62 (overlap, 3H, H1’b, H2, H5), 3.75 (br, 1H, H3), 4.63 (br, 1H, H1), 4.91 (overlap, 4H, H10', -OH), 5.00 (br, 1H, H10'), 5.79 (m, 1H, H9'). ^13^C-NMR (CD_3_OD): δ (ppm) 18.1 (C6), 27.4 (C2'), 30.1, 30.2, 30.5, 30.6, 30.7 (C3', C4', C5', C6', C7'), 34.9 (C8'), 68.5 (C1'), 69.7 (C5), 72.4 (C4), 72.5 (C3), 74.0 (C2), 101.6 (C1α), 115.0 (C10), 140.1 (C9’). Elemental analysis for C_16_H_30_O_5_, 0.25 H_2_O: calcd C: 62.62%; H: 10.02%. Found: C: 62.59%; H: 9.92%.

*1', 18'-bis-octadec-9'-enyl-α-L-rhamnopyranoside* (**3**)


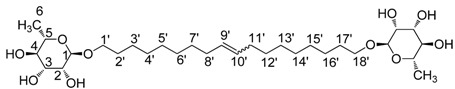


Compound **2** (2 g; 6.6 mmol; 1 eq) is diluted in CH_2_Cl_2_ (40 mL) in a Schlenk tube under argon and the Grubbs I catalyst (543 mg; 0.66 mmol; 0.1 eq) is added in three portions over 3 h. After 72 h of reaction at 40 °C, the solvent is evaporated under reduced pressure and the residue is purified by flash chromatography (eluting mixture: CH_2_Cl_2_/MeOH 9:1). Compound **3** is obtained as a brown wax with a yield of 21% as a mixture *Z/E* of 21/79. IR (KBr) ν cm^−1^: 3429 (F), 2926 (F), 2855 (m), 1634 (f), 1455 (f), 1131 (m), 1057 (F). ^1^H-NMR (CD_3_OD): δ (ppm) 1.24 (d, 6H, *J* = 5 Hz, H6), 1.31 (br, 20H, H3', H4', H5', H6', H7', H12', H13', H14', H15', H16’), 1.56 (br m, 4H, H2', H17’), 1.97 (br m, 2H, H8', H11'), 3.34 (overlap, 4H, H1'a, H18'a, H4), 3.62 (overlap, 6H, H1'b, H18'b, H2, H5), 3.76 (br, 2H, H3), 4.63 (br, 2H, H1), 4.87 (s, 6H, -OH), 5.37 (m, 2H, H9', H10'). ^13^C-NMR (CD_3_OD,): δ (ppm) 17.7 (C6), 26.9 (C3', C16'), 29.8–30.3 (C2', C4', C5', C6', C7', C12', C13', C14', C15', C16', C17'), 33.2 (C8',C11'), 68.1 (C1', C18'), 69.2 (C5), 71.8 (C4), 72.0 (C3), 73.5 (C2), 101.1 (C1α), 130.3 (C9'Z,C10'Z), 130.4, 131.0 (C9', C10'). Elemental analysis for C_30_H_56_O_10_, 1.1 H_2_O: calcd C: 60.40%; H: 9.83%. Found: C: 60.37%; H: 9.50%.

### 3.2. Physico-Chemical Characterization

Commercially-available dimethylsulfoxide (DMSO), trifluoroethanol (TFE) and deuterium oxide (D_2_O) at 99.9% isotopic purity were purchased from Sigma Chemical Co. (St. Louis, MO, USA). All solvents were analytical grade and used without further purification. The ultrapure water was produced by a Millipore system and had a resistivity of 18.2 MΩcm.

### 3.3. FTIR Spectroscopy

Infrared spectra were recorded by means of a Bruker Equinox 55 spectrometer (Karlsruhe, Germany) equipped with a liquid nitrogen-cooled Mercury-Cadmium-Telluride detector. The number scans was 128 and the resolution was 4 cm^−1^. All the experiments were performed with a demountable cell (Bruker) equipped with CaF_2_ windows [22]. During all experiments, the spectrophotometer was continuously purged with filtered dry air. The bolaamphiphile **3** was first dissolved in TFE at 10 mg/mL. The solution was then deposited on a CaF_2_ window and dried under vacuum in order to obtain a film. This resulting film was hydrated by spreading 10 µL of D_2_O. The spectrum of pure D_2_O was subtracted from the sample spectrum obtained under same conditions.

### 3.4. Adsorption Experiments at the Neat Air-Water Interface

The Wilhemy film balance was built by KSV (Helsinki, Finland) and placed on a vibration-isolated table. Bolaamphiphile **3** adsorption experiments to the neat air-water interface were performed in a KSV Minitrough with a volume of 190 cm^3^. The subphase was ultra-pure water at 25 °C and was continuously stirred with a magnetic spin stirrer at constant spinning. The bolaamphiphile **3** dissolved in DMSO was injected into the subphase. The adsorption was followed by measuring surface pressure as described previously [17]. The same volume of pure DMSO was injected under the lipid monolayer and no change of the surface pressure was detected.

### 3.5. Isothermal Titration Calorimetry (ITC) Experiments

Isothermal titration calorimetry was used in order to determine the thermodynamic parameters associated with the aggregation. Experiments were performed on a VP-ITC Microcalorimeter (Microcal, Northampton, MA, USA). The XylBol was dissolved in dimethylsulfoxide (DMSO) and the concentrated organic solution was diluted with milli-Q water (final DMSO concentration of 1% (v/v)). Aliquots of 0.27 mM solution of bolaamphiphile **1** (6 µL) were injected into a calorimeter cell at constant time intervals of 300s. The calorimeter cell had a volume of 1.4565 mL containing ultrapure water with 1% (v/v) of DMSO and its temperature maintained very accurately at 25 °C. The solution in the sample cell was stirred at a speed of 305 rpm. The reference cell was filled with milli-Q water. Prior to each analysis, all solutions were degassed using a sonicator bath. The heats related to the fall of the drop were determined by injecting at constant time interval 6 µL of water with 1% (v/v) of DMSO into the measuring cell containing water with 1% (v/v) of DMSO. These values were subtracted from the observed heats for determining the effective heats as described previously [10]. All measurements were repeated twice with two distinct bolaform solutions. Raw data were processed by software Origin 7 (Originlab, Northampton, MA, USA).

## 4. Conclusions

A new bolaamphiphile derived from an alkenyl l-rhamnoside was prepared easily in two reaction steps without recurring to a protection/deprotection procedure. The surface activity of this new bolaamphiphile was analyzed by Langmuir balance technology and compared with that of the analogous bolaamphiphile derived from alkenyl D-xylosides. The findings indicate the importance of the nature of the hydrophilic heads, the rhamnoside-based bolaform having an increased surface activity and a better ability to form aggregates than the xyloside-based one.
